# DKK-1 Is Underexpressed in Mesenchymal Stem Cells from Patients with Ankylosing Spondylitis and Further Downregulated by IL-17

**DOI:** 10.3390/ijms23126660

**Published:** 2022-06-15

**Authors:** Dimitrios Daoussis, Anastasia Kanellou, Elias Panagiotopoulos, Dionysios Papachristou

**Affiliations:** 1 Department of Rheumatology, University of Patras Medical School, Patras University Hospital, 26504 Patras, Greece; 2 Laboratory of Bone and Soft Tissue Studies, Department of Anatomy-Histology-Embryology, University of Patras Medical School, 26504 Patras, Greece; djpap70@gmail.com; 3 Department of Orthopedics, University of Patras Medical School, Patras University Hospital, 26504 Patras, Greece; ecpanagi@med.upatras.gr

**Keywords:** ankylosing spondylitis, new bone formation, Dickkopf-1, Dkk-1, IL-17, osteoblastogenesis

## Abstract

Dickkopf-1 (Dkk-1) is a key regulator of bone remodeling in spondyloarthropathies. Nevertheless, data regarding its expression in cells of pathophysiologic relevance, such as mesenchymal stem cells (MSCs), are lacking. Herein, we aimed to address DKK1 gene expression and Wnt pathway activation in MSCs from patients with ankylosing spondylitis (AS) and explore the effect of IL-17 on MSCs with respect to DKK-1 expression and Wnt pathway activation. Primary MSCs were isolated from the bone marrow of the femoral head of two patients with AS and two healthy controls undergoing orthopedic surgery. MSCs were cultured for 7 days in expansion medium and for 21 days in osteogenic medium in the presence or absence of IL-17A. Gene expression of DKK-1 and osteoblastic markers was determined by RT-PCR. Alkaline phosphatase activity, alizarin red and Van Kossa staining were used to assess osteoblastic function and mineralization capacity. DKK-1 was significantly downregulated in MSCs and osteoblasts from patients with AS compared to controls. Moreover, MSCs and osteoblasts from AS patients displayed increased Wnt pathway activation and enhanced osteoblastic activity, as indicated by increased expression of osteoblast marker genes and alkaline phosphatase activity. IL-17 downregulated DKK-1 expression and increased osteoblastic activity and mineralization capacity. DKK-1 is underexpressed in MSCs from AS patients compared to controls, whereas IL-17 has an inhibitory effect on DKK-1 expression and stimulates osteoblastic function. These data may have pathogenetic and clinical implications in AS.

## 1. Introduction

Ankylosing spondylitis (AS) is a chronic rheumatic disease characterized by inflammatory spinal pain and new bone formation, which gradually leads to ankylosis of the axial skeleton [1]. Mechanisms of new bone formation in the context of AS are not well understood. Nonetheless, a considerable amount of experimental evidence indicates that developmental pathways, such as the Wnt, hedgehog and bone morphogenetic protein pathways are crucially involved [2,3,4,5,6,7]. Most data implicate the Wnt pathway as a key driver of osteoblastogenesis/new bone formation [8,9]. The Wnt pathway is tightly regulated by several molecules, including the strong inhibitor Dickkopf-1 (Dkk-1) [10]. In animal models of arthritis, Dkk-1 has been shown to act as a master regulator of joint remodeling [11]. High Dkk-1 expression was linked to erosive disease; on the other hand, blockade of Dkk-1 led to osteophyte formation, completely reversing the joint remodeling process from an erosive phenotype towards a bone-forming phenotype. Further studies of TNF transgenic mice showed that Dkk-1 blockade led to Wnt pathway activation in sacroiliac joints and, subsequently, to ankylosis, providing strong experimental evidence that the Wnt pathway and specifically Dkk-1 are tightly linked to sacroiliac fusion/ankylosis [12]. In humans with AS, levels of Dkk-1 circulating in peripheral blood appear to be increased compared to controls [13]. Nevertheless, experimental evidence indicates that Dkk-1 may be dysfunctional in AS, as it fails to downregulate Wnt pathway activation in ex vivo experimental models [14]. Although Dkk-1 has been extensively studied as a soluble molecule, data are lacking regarding Dkk-1 expression in tissues of pathophysiologic relevance, such as bone or mesenchymal stem cells (MSCs), which are key players in new bone formation through the process of endochondral ossification [15].

In the last few years, there has been a substantial advance in the pathophysiology of spondyloarthropathies, with the establishment of the IL-23/IL-17 axis as a critical mediator [16]. IL-17 inhibitors have been approved for the treatment of AS and exhibit noteworthy clinical efficacy in terms of reducing spinal pain and increasing quality of life [17]. Clinical data indicate that radiographic progression under IL-17 blockers is very slow, although to date, there is no direct proof that these agents inhibit radiographic progression and modulate the natural course of the disease towards ankylosis [18]. Limited data are available regarding the role of IL-17 in the activation of the Wnt pathway and Dkk-1 expression.

Taking into account the crucial role of IL-17 in AS pathogenesis and experimental evidence suggesting that the Wnt pathway is strongly involved in new bone formation, in this study, we aimed to (i) assess DKK1 gene expression and Wnt pathway activation in MSCs from patients with AS compared to controls and (ii) explore the effect of IL-17 on MSCs with respect to DKK1 expression and Wnt pathway activation.

## 2. Results

MSCs and osteoblasts from AS patients underexpress DKK-1 and exhibit increased Wnt pathway activation and osteoblastic activity

**Figure 1:**
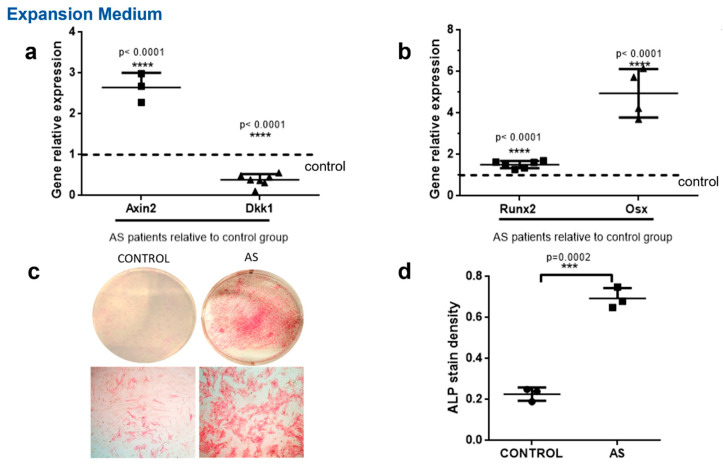
Decreased DKK1 expression and increased WNT pathway activity in MSCs from AS patients. MSCs from the control group and the AS group were cultured in vitro in expansion medium. (**a**,**b**) Axin2, Dkk1, Runx2 and Osx expression levels were determined with the quantitative RT-PCR method. Each gene’s expression is presented as a fold expression in AS cells relative to the control group expression normalized to unity. (**c**,**d**) ALP activity in cultured cells. (**c**) Macroscopic images of the culture dishes (top row) and representative microscope images (bottom row). (**d**) Scatter plots depicting the quantification of ALP stain density. Data are presented as mean ± SD. *** = *p* < 0.001; **** = *p* < 0.0001.

We first aimed to assess DKK-1 gene expression and Wnt pathway activity in MSCs from AS patients compared to healthy controls. We found that DKK-1 was significantly underexpressed in MSCs obtained from AS patients compared to healthy controls (*p* < 0.0001). Taking into account that Dkk-1 is a strong inhibitor of the Wnt pathway, we next assessed Wnt pathway activation by measuring the expression of the Wnt pathway target gene, Axin2. We observed a significant upregulation of Axin2 (*p* < 0.0001), indicating increased Wnt pathway activation in AS patients compared to controls (Figure 1a).

We next assessed whether increased Wnt pathway activation in AS MSC coincided with increased osteoblastic activity by evaluating the expression of the key osteoblastic genes, Runx2 and Osx, and ALP activity. Both Runx2 and Osx were upregulated in AS compared to control MSCs (*p* < 0.0001 for both Runx2 and Osx, Figure 1b). Moreover, AS MSCs presented with increased ALP activity compared to controls (*p* = 0.0002, Figure 1c,d).

**Figure 2:**
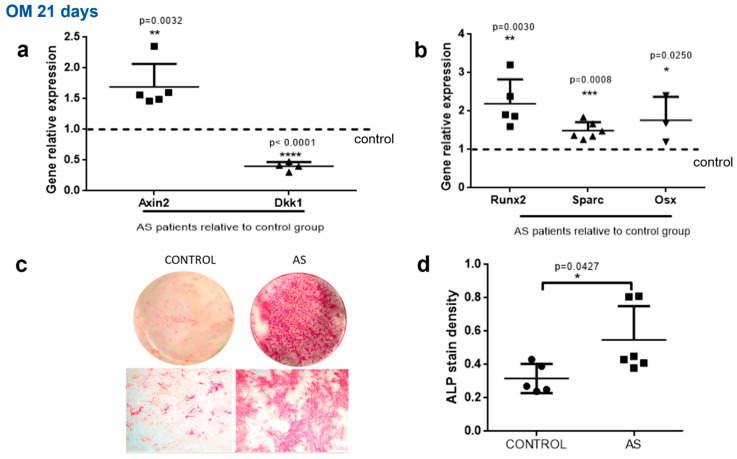
Decreased DKK1 expression and enhanced WNT pathway signaling and osteoblast differentiation in AS. MSCs from the control group and the AS group were cultured in vitro with osteogenic medium (OM) for 21 days. (**a**,**b**) Axin2, Dkk1, Runx2, Sparc and Osx expression levels were determined with the quantitative RT-PCR method. Each gene’s expression is presented as a fold expression in AS cells relative to the control group expression normalized to unity. (**c**,**d**) ALP activity in cultured cells. (**c**) Whole culture dish images (top row) and microscopic photographs (bottom row). (**d**) Scatter plots depicting the quantification of ALP stain density. Data are presented as mean ± SD. * = *p* < 0.05; ** = *p* < 0.01; *** = *p* < 0.001; **** = *p* < 0.0001.

Similar results were obtained when we analyzed MSCs committed to the osteoblastic lineage following culture in osteogenic medium. Osteoblasts from patients with AS exhibited significantly lower DKK-1 gene expression (*p* < 0.0001) and remarkably higher Axin2 expression (*p* = 0.0032) compared to controls (Figure 2a).

Increased Wnt pathway activation in AS osteoblasts coincided with enhanced osteoblastic activity, as shown by increased expression of the key osteoblastic target genes Runx2, SPARC and Osx (*p* = 0.003, *p* = 0.0008 and *p* = 0.025, respectively, Figure 2b). Moreover, ALP activity in AS osteoblasts was increased compared to controls (*p* = 0.042, Figure 2c,d). We observed a significant positive correlation between ALP activity and Runx2 expression (*p* = 0.023) and a trend toward positive correlation with Axin2 (*p* = 0.098).

Collectively, these data indicate that the expression of DKK-1 in MSCs from patients with AS is compromised and associated with augmented Wnt pathway activation and osteoblastic activity.

### IL-17 Further Downregulates DKK-1 in MSCs from AS Patients

**Figure 3:**
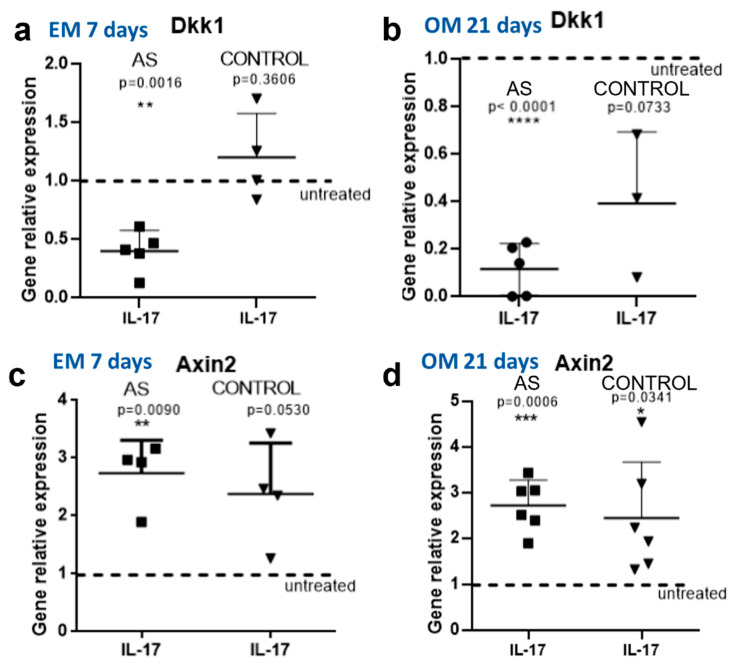
Effect of IL-17 on DKK1 expression and WNT pathway activation. DKK1 and Axin2 expression levels in the control group and AS group were determined by the quantitative RT-PCR method. (**a**) DKK1 relative expression following cell culture in expansion medium in the presence (IL-17) or absence (UT) of 50 ng/mL IL-17 for 7 days (EM 7 days) and (**b**) in osteogenic medium in the presence (IL-17) or absence (UT) of 50 ng/mL IL-17 for 21 days (OM 21 days). (**c**) Wnt activation marker Axin2 expression levels following cell culture in expansion medium in the presence (IL-17) or absence (UT) of 50 ng/mL IL-17 for 7 days (EM 7 days) and (**d**) in osteogenic medium in the presence (IL-17) or absence (UT) of 50 ng/mL IL-17 for 21 days (OM 21 days). All graphs indicate mean ± SD. Each assay is expressed as fraction of the UT expression normalized to unity. * = *p* < 0.05; ** = *p* < 0.01; *** = *p* < 0.001; **** = *p* < 0.0001.

We next focused on the effect of IL-17 on DKK-1 expression in MSC and osteoblasts from AS patients compared to controls. We found that IL-17 significantly downregulated the expression of DKK-1 genes in both MSCs (*p* = 0.0016, Figure 3a) and osteoblasts (*p* < 0.0001, Figure 3b). In sharp contrast, IL-17 had no effect on DKK-1 expression in control MSCs (*p* = 0.3606, Figure 3a), whereas in control osteoblasts, DKK-1 was downregulated but not to the level of statistical significance (*p* = 0.0733, Figure 3b).

By measuring the expression of Axin2, we found that IL-17 had a stimulatory effect on Wnt pathway activation in AS MSCs (*p* = 0.0090, Figure 3c). In control MSCs, there was a trend toward increased Axin2 expression, which did not reach the level of statistical significance (*p* = 0.0530, Figure 3c). A stimulatory effect of IL-17 was evident in both AS osteoblasts (*p* = 0.0006) and controls (*p* = 0.0341, Figure 3d). We additionally explored the effect of IL-17 on Osx and SPARC expression in osteoblasts. We observed a significant stimulatory effect of IL-17 on Osx expression in both AS (*p* = 0.0045) and controls (*p* = 0.044), whereas there was no significant effect on SPARC expression, as shown in Supplemental Figure S1.

**Figure 4:**
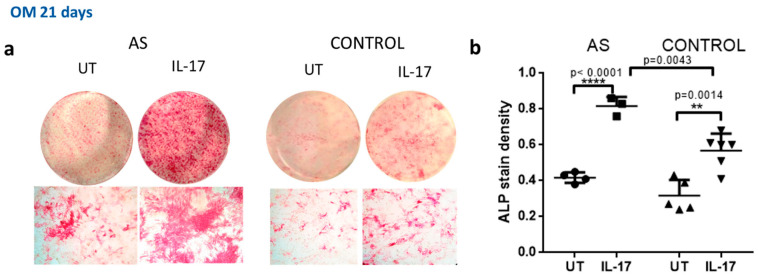
IL-17 enhances ALP activity. MSCs from the control group and AS group were cultured in osteogenic medium (OM) for 21 days in the presence (IL-17) or absence (UT) of 50 ng/mL IL-17, and ALP activity staining was performed. (**a**) ALP-stained representative photographs from whole culture dishes (top row) and microscopic fields (bottom row). (**b**) Quantification analysis of ALP stain density. Mean ± SD. ** = *p* < 0.01; **** = *p* < 0.0001.

We next analyzed the effect of IL-17 on the function of AS osteoblasts compared to controls by measuring ALP activity. Mineralization capacity was assessed with the use of alizarin red and Von Kossa staining. We found that IL-17 led to a significant increase in ALP activity in both AS (*p* < 0.0001 for treated vs. untreated cells (Figure 4a)) and control osteoblasts (*p* = 0.014 for treated vs. untreated cells (Figure 4b)).

**Figure 5:**
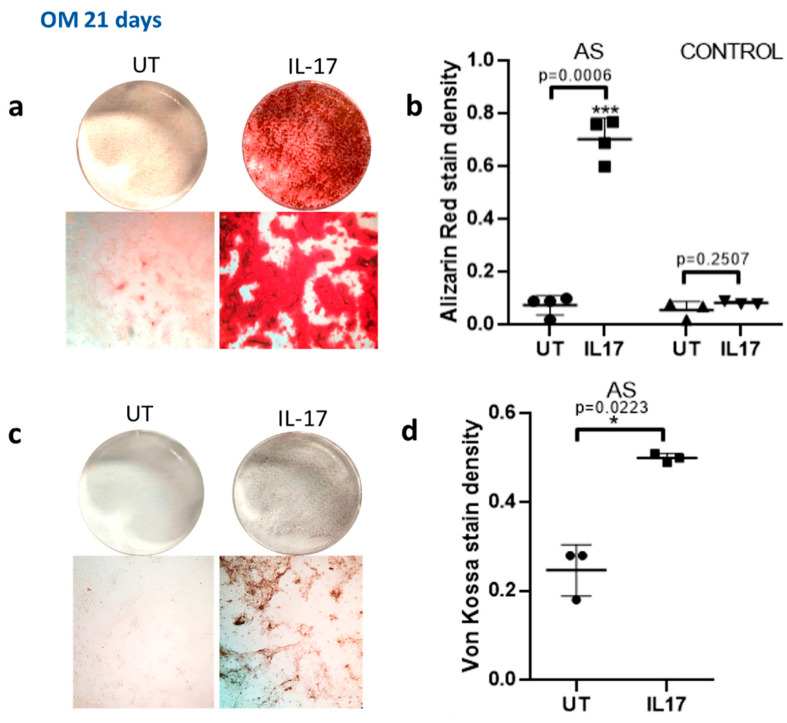
IL-17 induced mineralization in MSC cell cultures from AS group. MSCs were cultured in osteogenic medium (OM) for 21 days in the presence (IL-17) or absence (UT) of 50 ng/mL IL-17. (**a**) Alizarin red staining of AS cell cultures; macroscopic photographs (top row) and microscope images (bottom row). (**b**) Scatterplots depicting the quantification of Alizarin red stain density for AS and control group cell cultures. (**c**) Von Kossa staining of AS cell cultures; macroscopic photographs (top row) and microscope images (bottom row). (**d**) Densitometry analysis of Von Kossa stain. Mean ± SD. * = *p* < 0.05; *** = *p* < 0.001.

IL-17 enhanced mineralization capacity as assessed by alizarin red staining in AS osteoblasts (*p* = 0.0006 for treated vs. untreated cells) but not in controls (*p* = 0.2507, Figure 5a).

The enhancement of mineralization capacity in AS osteoblasts was verified by Von Kossa staining (*p* = 0.0223 for treated vs. untreated AS osteoblasts (Figure 5c,d)). Taken together, these data indicate that IL-17 has a suppressive effect on DKK-1 expression in MSCs from patients with AS, and this coincides with stimulation of osteoblastic activity and mineralization capacity.

## 3. Discussion

The first part of the current study was focused on assessing the expression of the Wnt pathway and its inhibitor, Dkk-1, in MSCs and committed osteoblasts from patients with AS compared to controls. Although in animal models, Dkk-1 acts as the master regulator of joint remodeling [11] and in humans with AS, evidence indicates that it is dysfunctional [14], the expression of this key molecule has not been assessed in human cells of major pathophysiological relevance, such as MSCs and osteoblasts. Our data show that MSCs from AS patients exhibit strikingly less Dkk-1 gene expression than controls. This reduced expression is associated with higher levels of Wnt pathway and ALP activity. Similar data were obtained in committed osteoblasts from patients with AS displaying lower DKK-1 expression and higher Wnt pathway activity. Committed osteoblasts from AS patients exhibited increased osteoblastic activity compared to controls. Taken together, these data indicate that MSCs and osteoblasts of patients with AS are activated and therefore more prone to new bone formation. The fact that DKK-1 downregulation is evident not only in MSCs but also in MSCs committed to the osteoblastic lineage following long-term ex vivo culture points towards an “intrinsic” defect rather than an effect of the AS inflammatory microenvironment [19]. This “intrinsic” activation, combined with immunologic or mechanical factors [20,21], may contribute to the bone-forming process in AS.

The second part of the study was focused on the effect of IL-17 on MSCs and osteoblasts from patients with AS. Although IL-17 is a well-known inducer of osteoclastogenesis [22,23], its effect on osteoblasts is not well understood. Studies of animal models have shown that IL-17 may have different effects on bone-forming cells depending on the stage of differentiation. Experimental evidence suggests that IL-17 induces osteoblast differentiation of MSCs in animal models [24,25,26]. On the other hand, IL-17 appears to downregulate bone-forming activity of committed osteoblasts [27,28,29].

Although it is known that IL-17 downregulates Dkk-1 expression in MSCs from healthy subjects [30], there is extremely limited evidence regarding the effect of IL-17 on human cells from patients with AS. A relatively recent study showed that IL-17 promotes osteoblastic differentiation of fibroblast-like synoviocytes derived from inflamed peripheral joints of patients with spondyloarthropathy [31]. Another study provided evidence that IL-17 had a stimulatory effect on primary bone-derived cells from patients with AS—a finding that is in line with our data [32]. Our study provides evidence that the stimulatory effect of IL-17 on MSCs may be mediated through DKK1 downregulation. Experimental evidence shows that IL-17 enhances osteogenic differentiation in periosteal cells, whereas IL-17 blockade increased DKK-1 expression and diminished new bone formation in an animal model [33].

Our data indicate that IL-17 has a stimulatory effect on MSCs by enhancing their differentiation toward osteoblasts and boosting their function and mineralization capacity. This effect may be at least partially mediated through DKK-1 downregulation. The effect of IL-17 inhibitors on new bone formation in patients with AS is not currently known. However, emerging data indicate that radiographic progression under IL-17 inhibition is rather slow. If this is verified in controlled studies, our data could provide mechanistic insight into how IL-17 inhibition may retard the rate of new bone formation.

However, our study has several limitations. The number of AS samples analyzed was small, therefore not allowing for definite conclusions to be drawn. Obtaining bone samples from AS patients is difficult and usually not feasible for research purposes. This is why in most cases, bone samples have been obtained during orthopedic interventions, as was in our case. Obtaining bone samples during orthopedic procedures is associated with another limitation; only samples from patients with long-term disease can be obtained, as only these patients require surgery. Another limitation of our study is that it does not address the question of why DKK-1 is downregulated in MSCs from patients with AS compared to controls. However, we hypothesize that post-transcriptional mechanisms may apply, as so far, no DKK1 gene single-nucleotide polymorphisms have been associated with either radiographic progression or Dkk-1 circulating levels [13,34]. Finally, a significant limitation is the fact that we were unable to complement our gene expression data with data regarding the expression of relevant proteins; moreover, detailed, functional experiments using RNA interference would provide more data. However, we were unable to do so due to the few samples available and the limited amount of cells. Our results could serve as a starting point for further larger-scale mechanistic studies. Among the strengths of our study is the fact that it is the first to assess DKK-1 expression in MSCs from AS patients, which are cells of pathophysiologic significance.

In conclusion, in the present study, we found that DKK-1 is underexpressed in MSCs from AS patients compared to controls, whereas IL-17 has an inhibitory effect on DKK-1 expression and stimulates osteoblastic function. These data may have pathogenetic and clinical implications and suggest that long-term use of IL-17 inhibitors may retard radiographic progression in AS.

## 4. Materials and Methods

### 4.1. Cell Isolation

Primary MSCs were isolated from the bone marrow of the femoral head of two patients with AS undergoing orthopedic surgery for joint replacement and two healthy controls with fractures. All donors provided written informed consent, and ethics committee approval was obtained (University Hospital of Patras Ethics Committee). Bone tissue was obtained under sterile conditions, and bone marrow cells from the trabecular bone were immediately suspended in alpha-MEM medium (Gibco, Invitrogen, Waltham, MA, USA) containing 100 U/mL penicillin–streptomycin (Gibco, Invitrogen). The cells were harvested by centrifugation (1350 rpm, 5 min, 20 °C), and the erythrocytes were lysed. The remaining cells were seeded at 2 × 10^4^/cm^2^, suspended in expansion medium (EM) composed of alpha-MEM medium, 10% fetal bovine serum (FBS, Gibco, Invitrogen, Waltham, MA, USA) and 100 U/mL penicillin–streptomycin and maintained at 37 °C in a humidified environment of 5% CO_2_. The non-adherent cells were removed after the first medium change two days later. Cells were used for the experiments between passages 1 and 2. Cells derived from different donors were never pooled.

### 4.2. In Vitro IL-17 Stimulation

In vitro IL-17 simulation of the MSCs was conducted according to two distinct protocols.

The MSCs were cultured for 7 days in EM in the presence or absence of 50 ng/mL recombinant human IL-17A (R&D Systems, Minneapolis, MN, USA).

For the differentiation protocol, MSCs were cultured for 21 days in osteogenic medium (OM) containing DMEM:F12 (Gibco, Invitrogen), 15% FBS, 100 U/mL penicillin–streptomycin, 100 nM dexamethasone (Sigma, St. Louis, MO, USA), 5 mM β-glycerophosphate (Sigma) and 50 μg/mL ascorbic acid (Sigma) in the presence or absence of 50 ng/mL recombinant IL-17A (R&D Systems). The medium was replaced every 3–4 days.

### 4.3. Quantitative RT-PCR Analysis

For quantitative RT-PCR analysis, total RNA was extracted from the cultured cells using a NucleoSpin RNA kit (Macherey-Nagel, Wilmington, MA, USA), and cDNA was synthesized with a PrimeScript first-strand cDNA synthesis kit (Takara, San Jose, CA, USA) from 1 μg total RNA. RT-PCR was performed on a MX3000PTM Quantitative PCR system thermal cycler (Stratagene, La Jolla, CA, USA) using a KAPA SYBR FAST Universal qPCR kit (Kapa Biosystems, Allentown, PA, USA). A list of primers used is shown in Table 1.

The relative mRNA expression of genes was calculated using the 2^−ΔΔCT^ method. The expression of target genes was normalized to the expression of GAPDH. Each reaction was performed in triplicate.

### 4.4. Alkaline Phosphatase (ALP) Staining

Alkaline phosphatase is a commonly used marker for early osteoblastic differentiation. To assess alkaline phosphatase activity, cells were fixed with cold 10% neutral formalin buffer for 15 min. After rinsing with dH_2_O, cells were incubated for 45 min at room temperature with 0.1 mg/mL naphthol AS-MX phosphate (Sigma) substrate in 0.4% N,N-dimethylformamide and 0.1 M HCl (pH 8.3) with 0.6 mg/mL fast red violet LB salt (Sigma) to visualize the product. The staining solution was washed away with dH2O. The products of the enzyme activity were detected as red stains.

### 4.5. Alizarin Red Staining

The cells cultured with osteogenic medium were stained with Alizarin Red stain (AR) to examine matrix mineralization. Cells were fixed with cold 10% neutral formalin buffer for 30 min. Then, they were stained for 4 min at room temperature with alizarin red stain (pH: 4.2, Sigma). Alizarin red dyes calcium deposits with red color.

### 4.6. Von Kossa Staining

For the assessment of matrix mineralization, cells cultured with osteogenic medium were also stained by the Von Kossa staining (VK) method. After fixation with 10% neutral formalin buffer for 30 min, cells were treated with 1% silver nitrate solution for 60 min under strong light. The mineral depositions obtained a metallic silver color. The stains were visible to naked eye and under a light microscope. The stained area was quantified using Image J software.

Digital images were obtained from different areas of each dish (objective ×5) and analyzed with Image J software (Java-based image-processing program, NIH) using the “grid’’ tool.

### 4.7. Statistical Analysis

Analysis was performed using the two-tailed Student’s *t*-test. Differences with *p* < 0.05 were considered significant. Data are represented as mean ± S.D. GraphPad Prism version 6 (GraphPad Software, San Diego, CA, USA) was used to analyze and present the reported data.

## Figures and Tables

**Table 1:** List of primers used for RT-PCR analysis.

Genes	Forward Primer	Reverse Primer
Runx2	5′-CGGAGTGGACGAGGCAAGA-3′	5′-GAGGCGGTCAGAGAACAAACT-3′
Osterix	5′-AGGTTCCCCCAGCTCTCT-3′	5′-TTCTTTGTGCCTGCTTTGCC-3′
Osteonectin (sparc)	5′-GGCAGAGGTGACTGAGGTATC-3′	5′-GTTTGCAGTGGTGGTTCTGG-3′
Axin2	5′-TGTGAGGTCCACGGAAACTG-3′	5′-TTCCATCTACACTGCTGTCCG-3′
Dkk1	5′-GAATAAGTACCAGACCATTGAC-3′	5′-CCATTTTTGCAGTAATTCCC-3′

## Data Availability

All data are available upon reasonable request from corresponding authors.

## Supplementary Materials

The following supporting information can be downloaded at: https://www.mdpi.com/article/10.3390/ijms23126660/s1.
