# Supplementary material for: DKK-1 Is Underexpressed in Mesenchymal Stem Cells from Patients with Ankylosing Spondylitis and Further Downregulated by IL-17
Source: Int J Mol Sci. 2022 Jun 15;23(12):6660. doi: 10.3390/ijms23126660 (PMC9224314; doi:10.3390/ijms23126660)
Supplement: Supplementary file 1 [file ijms-23-06660-s001.zip › ijms-1653453-supplementary.pdf]

Supplemental Figure S1

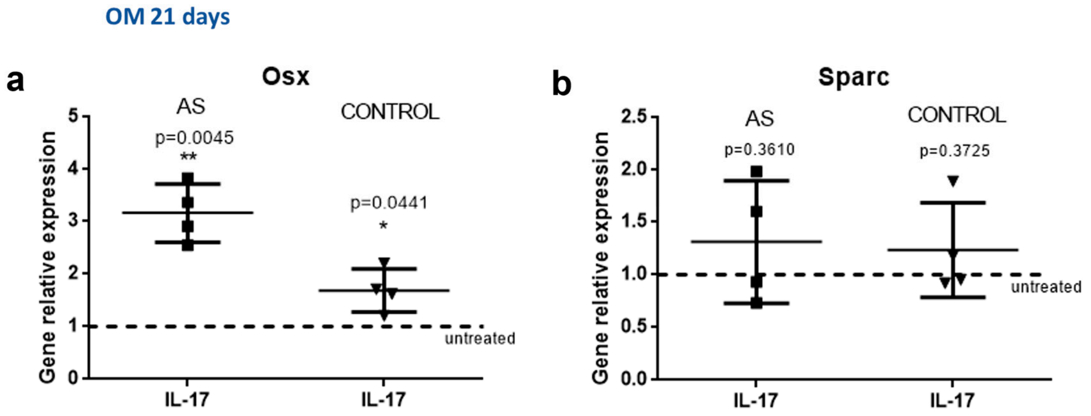

Figure S1: IL-17 promotes the expression of osteoblastic markers in MSC cell cultures. MSCs from the control group and AS group were cultured in osteogenic medium (OM) for 21 days in the presence (IL-17) or absence (UT) of 50 ng/mL IL-17. Scatter plots indicate **(a)** Osx and **(b)** Sparc relative expression levels as they were determined with quantitative RT-PCR method. Each gene's expression is presented as a fold expression in IL-17 treated cells relatively to the UT expression normalized to unity (horizontal line). Data are represented as mean ± SD. * = p<0.05; ** = p<0.01.
